# RNA-Seq reveal the circular RNAs landscape of lung cancer

**DOI:** 10.1186/s12943-019-1118-8

**Published:** 2019-12-12

**Authors:** Christophe Nicot

**Affiliations:** Department of Pathology and Laboratory Medicine, Center for Viral Pathogenesis, University of Kansas Medical Center, 3901 Rainbow Boulevard, Kansas City, KS 66160 USA

Few studies have used high-throughput RNA sequencing for functional characterization of patients with non-small cell lung cancer (NSCLC) including lung adenocarcinoma (LUAD) and lung squamous cell carcinoma (LUSC). In a study recently published in *Molecular Cancer*, Wang and colleagues’ work revealed the common and histopathological subtype-specific circular RNA (circRNA) expression patterns between LUAD and LUSC and highlighted the important diagnostic potential of circRNAs in lung cancer [1].

To obtain a comprehensive and accurate quantification of circRNAs, the authors analyzed high-throughput RNA sequencing data from 10 pairs of lung tumors and their adjacent normal tissues using five computational tools. Results revealed 17,952 circRNAs including 2593 previously unidentified circRNAs. Using stringent criteria (corrected *p*-value ≤0.05 and fold change ≥2), differential expression of 50 and 172 circRNAs was identified in tumor tissues of LUAD and LUSC, respectively. In addition, 26 circRNAs were commonly deregulated in both tumors. Moreover, another independent cohort of 67 lung cancer patients’ samples were subjected to experimentally validation of RNA-sequencing data. Results were consistent and validated the accuracy and robustness of the circRNA analysis pipeline.

The authors also conducted the Receiver Operating Characteristic (ROC) curve analysis of several selected circRNAs and revealed high diagnostic potential of hsa_circ_0077837 and hsa_circ_0001821 in discriminating NSCLC from normal tissues. Authors’ results also highlighted the potential application of hsa_circ_0001073 and hsa_circ_0001495 for diagnostic in the histopathological subtyping of NSCLC. Collectively, this study demonstrated that LUAD and LUSC as the major subtype of NSCLC share a common differential expression pattern, but also have their unique circRNA expression signatures. Identified circRNAs may serve as and new clinical tools for diagnosis of pathological subtypes of NSCLC and therapeutic intervention.

## Data Availability

Not applicable
